# BAY-885, a mitogen-activated protein kinase kinase 5 inhibitor, induces apoptosis by regulating the endoplasmic reticulum stress/Mcl-1/Bim pathway in breast cancer cells

**DOI:** 10.1080/21655979.2022.2078557

**Published:** 2022-05-24

**Authors:** Lei Wang, Xiaochun Ji, Chenxiao Mao, Rui Yu

**Affiliations:** a Department of Thyroid and Breast Surgery, Ningbo Medical Centre, the Affiliated Lihuili Hospital of Ningbo University, Ningbo; b Department of Electronic Commerce, Zhejiang Fashion Institute of Technology, Ningbo; c Department of Biochemistry, School of Medicine, Ningbo University, Ningbo

**Keywords:** Breast cancer, BAY-885, ERK5, mcl-1

## Abstract

The mitogen-activated protein kinase kinase 5 (MEK5)/extracellular signal-regulated kinase 5 (ERK5) axis has been reported to promote tumorigenesis in breast cancer (BC). Therefore, targeting the MEK5/ERK5 axis is a potential strategy against BC. BAY-885 is a novel inhibitor of ERK5; however, to date, its anti-tumor effects in BC have not been investigated. This study aimed to assess the anti-tumor effects of BAY-885 in BC and identify its underlying mechanisms of action. Unlike other ERK5 inhibitors, which frequently failed to mimic ERK5 genetic ablation phenotypes, the BAY-885 treatment effectively recapitulated ERK5 depletion effects in BC cells. Results revealed that BAY-885 affected the viability and induced apoptosis in BC cells. Moreover, the BAY-885-mediated downregulation of myeloid cell leukemia-1 (Mcl-1) and upregulation of Bim were dependent on ERK5 inhibition. Furthermore, BAY-885 triggered activation of endoplasmic reticulum (ER) stress, which further led to the upregulation of Bim and downregulation of Mcl-1. ER stress was induced in an ERK5 inhibition-dependent manner. These findings suggested that BAY-885 induced apoptosis in BC cells via ER stress/Mcl-1/Bim axis, suggesting that BAY-885 may serve as a therapeutic agent for BC.

## GRAPHICAL ABSTRACT

**Figure:**
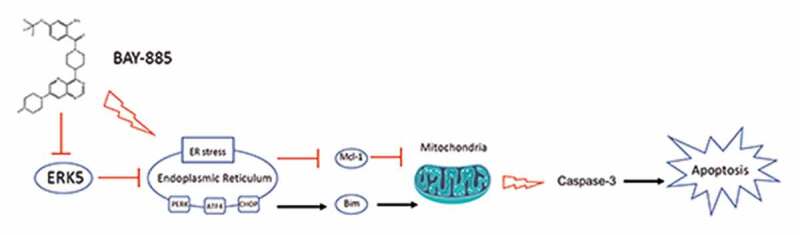

## Highlights

- BAY-885 showed selectively toxicity against BC cells but spare the normal cells
- BAY-885 treatment caused apoptosis via the mitochondrial pathway in BC cells
- BAY-885 treatment induced ER stress which further caused downregulation of Mcl-1 and upregulation of Bim

## 1. Introduction

Breast cancer (BC) is the leading cause of cancer-related deaths among women, and its incidence has increased substantially over the years [1]. Approximately more than 1.3 million new cases of BC and 0.5 million BC-related deaths occur annually worldwide [1]. Currently, surgery, chemotherapy, and radiotherapy are the principal strategies for the treatment of patients with BC. However, the efficacy of these methods is often limited because of many factors, such as drug resistance and distant metastasis [1]. Therefore, to improve patient outcomes, it is necessary to identify novel therapeutics for BC.

The mitogen-activated protein kinase (MAPK) signaling pathway is involved in various physiological activities such as differentiation, migration, and development of cancers [2]. To date, four types of MAPKs have been reported: c-Jun N-terminal kinase (JNK), p38, extracellular regulated kinases 1/2 (ERK1/2), and extracellular regulated kinases 5 (ERK5). Among them, ERK5, which is the least studied, can be activated by the upstream kinase MEK5 [3]. Evidence suggests that the MEK5/ERK5 axis plays an essential role in BC tumorigenesis; for example, ERK5 activation promotes the growth and maintenance of BC cells via regulation of the extracellular matrix [4]. Another study reported that upregulation of MEK5 promoted invasion and metastasis of BC cells [5]. Additionally, the MEK5/ERK5 axis is associated with poor survival of patients with BC after systemic treatments [6]. Thus, targeting the MEK5/ERK5 axis might be a potential strategy to treat BC.

BAY-885 is a novel, potent, and selective inhibitor of ERK5 [7]. To date, the anti-tumor effects of BAY-885 have not been studied in detail. This study aimed to investigate the effects of BAY-885 on BC cells and identify its underlying mechanisms of action. BAY-885 toxicity was found to be selectively harmful to BC cells but not to normal cells. Unlike other ERK5 inhibitors, which frequently failed to mimic ERK5 genetic ablation phenotypes.BAY-885 treatment could recapitulate ERK5 depletion effects, and genetically-induced activation of constitutively activated ERK5 abrogated the effects of BAY-885 in BC cells. Thus, BAY-885 is a potent and specific ERK5 inhibitor. Considering that ERK5 is frequently overexpressed and activated in patients with BC and linked with a poor clinical outcome [8], our results suggest that BAY-885 can be a promising therapeutic agent against BC.

## 2. Materials and methods

### 2.1 Chemicals and cell culture

BAY-885 and epirubicin were obtained from Selleck Chemicals (USA). Doxorubicin, trastuzumab, and paclitaxel were generous gifts from Dr. Yu Ren (Ningbo Yinzhou No. 2 Hospital). All other chemicals were purchased from Sigma-Aldrich (USA) unless specified elsewhere. BC cell lines and human mammary epithelial cells (MCF10A) were obtained from American Type Culture Collection (ATCC, USA). All cells were maintained in RPMI1640 medium (Gibco, USA) supplemented with 10% fetal bovine serum (FBS, Gibco) and 1% penicillin/streptomycin (Sigma, USA). Cells were maintained in a humidified incubator with 5% CO_2_ at 37°C.

### 2.2 Cell viability assay

Cell viability was measured using the Cell Counting Kit-8 (CCK-8, Donjido, Japan), according to the manufacturer’s protocol. Briefly, cells were seeded into a 96-well plate at a density of 5000 cells/well. After different treatments, 10 μL of the CCK-8 solution was added to each well. Then, the plates were incubated for 2 h at 37°C. The absorbance of the cells at 450 nm was recorded by a microplate reader (BioTek, USA). Each experiment was conducted in triplicate and repeated at least three times.

### 2.3 Cell death assessment

The Annexin-V and propidium iodide (PI) staining kit (BD bioscience) was used to assess cell death according to the manufacturer’s protocol. In brief, cells were seeded into a 6-well plate at a density of 1 × 106 cells/well. After different treatments, cells were collected and stained with 5 μL of FITC-Annexin V and PI solution for 30 min at room temperature, in the dark. The number of positively stained cells was measured using FACSCalibur (BD Bioscience, USA). Caspase cleavage was assessed as an additional indicator of apoptosis.

### 2.4 Cell transfection

For Bim knockdown, siRNA against Bim (si-Bim) and scramble negative control (si-NC) were obtained from RioBio Technology (China). The siRNA sequence was as followings: si-Bim: 5’-UCUUACGACUGUUACGUUAUUdTdT-3′ (sense) and 5′-pUAACGUAACAGUCGUAAGAUUdTdT-3′ (antisense). si-NC: pGGCAAGCACCCUGAAGUUCUUdTdT-3′ (sense) and 5′-pGAACUUCAGGGUCAGCUUGCCUUdTdT-3′ (antisense). Vectors expressing a constitutively activated form of MEK5 (MEK5DD) or Mcl-1 were purchased from GenePharma Ltd. (China). Transfection was performed using Lipofectamine 2000 (Life Technologies, USA) according to the manufacturer’s protocol. Cells were incubated with siRNAs for 4 h, and the medium was replaced with fresh medium.

### 2.5 RT-PCR

Total RNA was extracted using TRIzol reagent (Life Technologies, USA) and reverse transcribed into cDNA using the PrimeScript RT Master Mix (Takara, China), following the manufacturer’s instructions. RT-PCR was performed on the CFX Connect^TM^ Real-Time System (Bio-Rad, USA). The primers used were ordered from GenePharma (China). PCR conditions were as follows: initial denaturation at 95°C for 30s followed by 35 cycles at 95°C for 5 s and 60°C for 20s. The mRNA expression of the target gene was normalized to that of GAPDH using the 2^−ΔΔCt^ method [9]. The following primers were used in this study: Mcl-1: (F) GGACATCAAAAACGAAGACG and (R) GCAGCTTTCTTGGTTTATGG; GAPDH: (F) CCACATCGCTCAGACACCAT and (R) CCAGGCGCCCAATACG.

### 2.6 RNA sequencing

RNA sequencing was conducted by Shanghai Bohao Biotechnology (China). Briefly, cells were treated with BAY-885 (2 μM) for 6 h. They were further purified and analyzed to construct libraries. Markedly differentially expressed genes were determined using edgeR software, and functional analysis was determined by Gene Ontology (GO) and Kyoto Encyclopedia of Genes and Genomes (KEGG) pathway.

### 2.7 Western blot analysis

Total proteins were separated using 5%–12% sodium dodecyl sulfate–polyacrylamide gel electrophoresis. Further, they were transferred onto a polyvinylidene fluoride membrane and blocked with 5% skimmed milk for 1 h at 25°C. The membrane was incubated with primary antibodies overnight at 4°C and subsequently with the secondary antibody for 1 h at room temperature. The blot was visualized using the enhanced chemiluminescence kit (Life Technologies, USA) on the ImageLadTM platform (BioRad, USA). The following antibodies were used in this study: p-ERK5 (cat:3371, dilution: 1:1000; Cell Signaling Technology), ERK5 (cat:3372, dilution: 1:1000; Cell Signaling Technology), Bcl-2 (cat:15,071, dilution: 1:1000; Cell Signaling Technology), Bim (cat: 2819, dilution: 1:1000; Cell Signaling Technology), Bcl-xl (cat: 2762,dilution: 1:1000; Cell Signaling Technology), Mcl-1 (cat: 4572,dilution: 1:1000; Cell Signaling Technology), p-Mcl-1 (cat: 14,765,dilution: 1:1000; Cell Signaling Technology), p-PERK (cat: 3179,dilution: 1:1000; Cell Signaling Technology), PERK (cat: 5683,dilution: 1:1000; Cell Signaling Technology), ATF4 (cat: 11,815,dilution: 1:1000; Cell Signaling Technology), CHOP (cat: 2895,dilution: 1:1000; Cell Signaling Technology), Caspase-3 (cat: 9662,dilution: 1:1000; Cell Signaling Technology), GAPDH (cat: 5174,dilution: 1:10,000; Cell Signaling Technology), HRP-conjugated anti-mouse (cat: 43,593, dilution: 1:5000, Cell Signaling Technology) or anti-rabbit secondary antibodies (cat:7074, dilution: 1:5000, Cell Signaling Technology)

### 2.8 Statistical analysis

Statistical analyses were performed using SPSS 12.0 (IBM, Chicago, IL, USA). Data were expressed as the mean ± standard deviation. The student’s t-test was used to compare differences between two groups, and one-way analysis of variance was used for comparisons among multiple groups, followed by Tukey’s post hoc test. Statistical significance was set at P < 0.05 (two-tailed).

## 3. Results

This study aims to unveil the effects of BAY-885 on BC cells. We assumed that BAY-885 could exerts anti-tumor effects against BC cells. First, we examined the effect of BAY-885 on cell viability and cell death of BC cell lines. In order to examine the molecular mechanism, the expression of apoptosis-related proteins was tested. In addition, the effect of BAY-885 on ER stress signaling markers was also evaluated. Genetic knockdown/overexpression and pharmacological inhibition were applied to verify the role of proteins/pathways involved in the anti-tumor effects of BAY-885 in BC cells.

### 3.1 BAY-885 inhibited viability and induced apoptosis in BC cells

**Figure 1.**
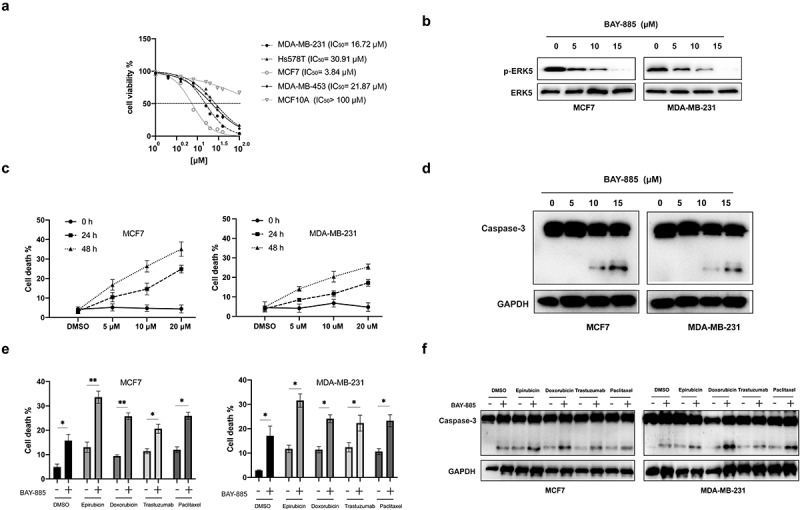
BAY-885 inhibited viability and induced apoptosis in breast cancer (BC) cells.

To evaluate the anti-tumor effects of BAY-885, BC cell lines (MCF7, MDA-MB-231, Hs578T, and MDA-MB-453) and human mammary epithelial cells (MCF10A) were treated with various doses of BAY-885 for 24 h, and cell viability was measured by CCK-8 assay. BAY-885 inhibited the viability of BC cells in a dose-dependent manner (Figure 1a). Interestingly, BAY-885 slightly inhibited the viability of MCF10A cells (IC50 > 100 μM) (Figure 1a). Because MCF-7 (IC50 = 3.84 μM) and MDA-MB-231 (IC50 = 30.91 μM) cells were the most sensitive and insensitive cell lines to BAY-885, respectively, they were subjected to further experiments. Western blot analysis showed that BAY-885 successfully inhibited p-ERK5 expression early at 1 h post-treatment in both MCF-7 and MDA-MB-231 cells (Figure 1b). Furthermore, MCF-7 and MDA-MB-231 cells were treated with various doses of BAY-885 (5, 10, and 20 μM) for different periods (24 and 48 h), after which cell death was analyzed. Interestingly, BAY-885 induced the death of both BC cell lines in a time- and dose-dependent manner (Figure 1c). Western blot analysis showed that BAY-885 induced activation of caspase-3 in BC cells in a dose-dependent manner (Figure 1d). Furthermore, BC cells were treated with BAY-885 (10 μM) in combination with other chemotherapeutic agents (epirubicin 10 μM, doxorubicin 5 μM, trastuzumab 20 μM, or paclitaxel 15 μM) for 24 h, after which cell viability was measured. BAY-885 increased the apoptosis induced by various chemotherapeutic agents in BC cells (Figures 1e and f). Collectively, these data suggested that BAY-885 exerted selective cytotoxicity against BC cells.

A. BC and normal cells were treated with various concentrations of BAY-885 for 24 h, and cell viability was measured by the CCK-8 assay. B. MCF-7 and MDA-MB-231 cells were treated with BAY-885 (10 μM) for the indicated times, and ERK5 levels were measured using western blot. C. MCF-7 and MDA-MB-231 cells were treated with the indicated doses of BAY-885 for the indicated times, and cell viability was measured by the CCK-8 assay. D. MCF-7 and MDA-MB-231 cells were treated with the indicated doses of BAY-885 for 24 h, and protein expression was measured using western blot. E. MCF-7 and MDA-MB-231 cells were treated with BAY-885 alone or in combination with various chemotherapeutic agents for 24 h, and cell viability was measured. F. MCF-7 and MDA-MB-231 cells were treated with BAY-885 alone or in combination with various chemotherapeutic agents for 24 h, and protein expression was measured using western blot. Data are representative of at least three independent experiments; *P < 0.05; **P < 0.01.

### 3.2 BAY-885 induced apoptosis of BC cells by regulating Mcl-1 and Bim expression

**Figure 2.**
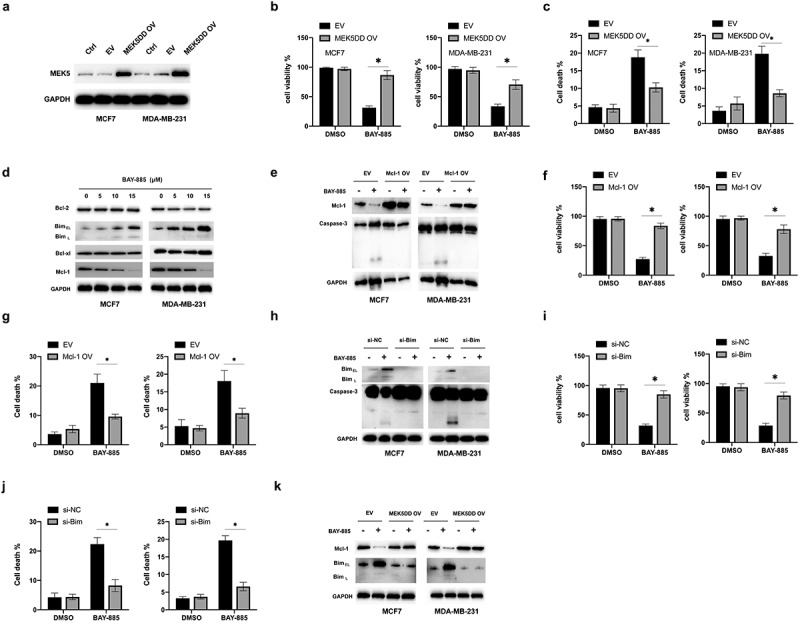
BAY-885-induced apoptosis in breast cancer (BC) cells in an ERK5 inhibition-dependent manner.

To assess whether the anti-tumor effects of BAY-885 were dependent on ERK5 inhibition, a vector expressing a constitutively activated form of MEK5 (MEK5DD) was transfected into BC cells (Figure 2a). Overexpression of MEK5DD significantly increased the viability of BC cells under BAY-885 treatment (Figure 2b). Meanwhile, forced expression of MEK5DD decreased the apoptosis caused by BAY-885 in BC cells (Figure 2c). Therefore, blockade of ERK5 was found to be critical for the anti-tumor effects of BAY-885 in BC cells. Since apoptosis is strictly subjected to the regulation of various Bcl-2 family members, we examined the effects of BAY-885 on changes in the expression of Bcl-2 family members. BAY-885 treatment led to the downregulation of Mcl-1 and upregulation of Bim, whereas other Bcl-2 proteins were not affected (Figure 2d). To investigate the role of Mcl-1 in the BAY-885-mediated apoptosis of BC cells, a vector expressing Mcl-1 was transfected into BC cells. Forced expression of Mcl-1 inhibited the cleavage of caspase-3 caused by BAY-885 in BC cells (Figure 2e). Simultaneously, overexpression of Mcl-1 increased the cell viability and decreased the apoptosis of BAY-885-treated BC cells (Figures 2 F and g). Furthermore, the cytotoxic role of Bim in BC cells under BAY-885 treatment was investigated by silencing Bim expression. Knockdown of Bim decreased the cleavage of caspase-3 caused by BAY-885 in BC cells (Figure 2h). Additionally, it promoted cell viability and decreased apoptosis of BC cells under BAY-885 treatment (Figures 2 I and j). Interestingly, overexpression of MEK5DD abrogated the effects of BAY-885 on Mcl-1 and Bim expression. Collectively, these data suggested that BAY-885 regulated the expression of Mcl-1 and Bim, both of which are critical for the BAY-885-mediated cytotoxicity, in an ERK5 inhibition-dependent manner.

A. MCF-7 and MDA-MB-231 cells were transfected with an empty vector (EV) or vector expressing MEK5DD (MEK5DD OV) for 24 h. Next, the cells were treated with or without BAY-885 for another 24 h. Cellular lysates were subjected to western blot analysis with the indicated antibodies. B. MCF-7 and MDA-MB-231 cells were treated as described in Figure 1. C. Cell viability was measured. D. MCF-7 and MDA-MB-231 cells were treated with the indicated doses of BAY-885 for 24 h. E. MCF-7 and MDA-MB-231 cells were transfected with EV or vector expressing Mcl-1 (Mcl-1 OV) for 24 h. Furthermore, the cells were treated with or without BAY-885 for another 24 h. G. Cell viability was measured. H. MCF-7 and MDA-MB-231 cells were transfected with si-NC or si-Bim for 24 h and further treated with or without BAY-885 for another 24 h. Cellular lysates were subjected to western blot analysis with the indicated antibodies. I. Cell viability was measured. J. Cell death was measured. K. MCF-7 and MDA-MB-231 cells were transfected with EV or MEK5DD OV for 24 h. Furthermore, the cells were treated with or without BAY-885 for another 24 h, and levels of Mcl-1 and Bim were measured using western blot analysis. Data are representative of at least three independent experiments; *P < 0.05; **P < 0.01.

### 3.3 BAY-885 activated the endoplasmic reticulum stress signaling pathway in BC cells

**Figure 3.**
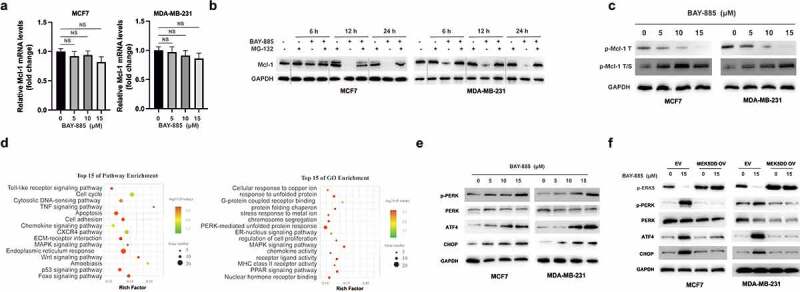
BAY-885 induced endoplasmic reticulum (ER) stress in breast cancer (BC) cells.

To examine the mechanism underlying Mcl-1 downregulation, we first performed RT-PCR analysis, which indicated that BAY-885 did not markedly affect *Mcl-1* mRNA levels (Figure 3a). Meanwhile, the decrease in Mcl-1 expression was prevented by MG-132, a proteasome inhibitor (Figure 3b). Thus, we postulated that Mcl-1 might be regulated by post-translation modifications. Although T163 phosphorylation stabilizes Mcl-1, dual T163/S159 phosphorylation primes Mcl-1 to be for proteasomal degradation [10]. As shown in Figure 3c, BAY-885 treatment inhibited T163 phosphorylation and increased dual T163/S159 phosphorylation of Mcl-1 in BC cells (Figure 3c).

A. MCF-7 and MDA-MB-231 cells were treated with various doses of BAY-885 for 24 h, and mRNA levels of *Mcl-1* were measured by RT-PCR. B. BC cells were treated with BAY-885 (10 μM), MG-132 (50 μM), or their combination for the indicated times. Mcl-1 levels were measured by western blot analysis. C. BC cells were treated with the indicated doses of BAY-885 for 24 h, and Mcl-1 phosphorylation status was assessed by western blot analysis. D. GO and KEGG analysis showed that Mcl-1 was enriched in URP and ER stress pathways. E. MCF-7 and MDA-MB-231 cells were treated with the indicated doses of BAY-885 for 24 h, and the expression of the indicated proteins was measured using western blot analysis. F. MCF-7 and MDA-MB-231 cells were transfected with EV or MEK5DD OV for 24 h. Furthermore, the cells were treated with or without BAY-885 for another 24 h, and cellular lysates were subjected to western blot analysis with the indicated antibodies. Data are representative of at least three independent experiments; *P < 0.05; **P < 0.01.

To further investigate the underlying mechanism of action of BAY-885 in BC cells, RNA sequencing was conducted in MCF-7 cells. Gene Ontology (GO) and Kyoto Encyclopedia of Genes and Genomes (KEGG) enrichment analyses showed the enrichment of ER stress (gene number, 18; P < 0.01) and unfolded protein response (UPR; gene number, 13; P < 0.01) signaling pathways (Figure 3d). To verify this, western blot analysis was performed, and the results showed that BAY-885 treatment led to an increase in the expression of p-PERK, ATF4, and CHOP in a dose-dependent manner in BC cells (Figure 3e). Moreover, overexpression of MEK5DD inhibited the activation of the ER stress signaling pathway caused by BAY-885 in BC cells (figure 3f). Collectively, these data suggested that BAY-885 activated the ER stress signaling pathway in an ERK5 inhibition-dependent manner in BC cells.

### 3.4 Blockage of ER stress abrogated the effects of BAY-885 on Mcl-1 and Bim expression

**Figure 4.**
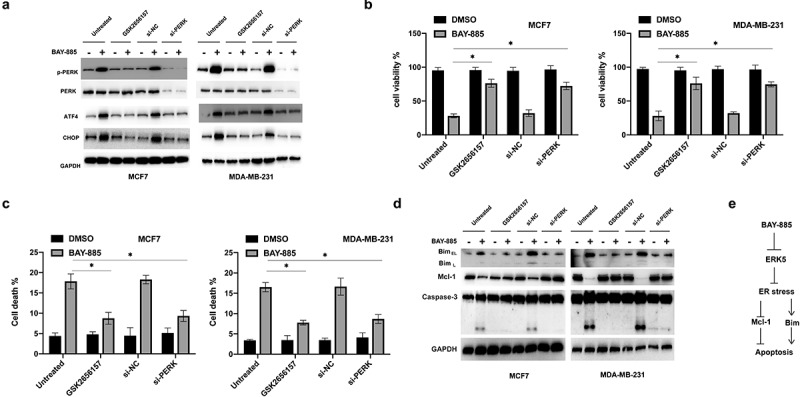
BAY-885 induced endoplasmic reticulum (ER) stress in an ERK5 inhibition-dependent manner.

Furthermore, we investigated whether there is any correlation between ER stress and change in the levels of Mcl-1 and Bim expression. The ER stress inhibitor GSK2656157 and si-PERK were used to inhibit ER stress signaling BAY-885-treated BC cells (Figure 4a). Results of the cell viability assay showed that either administration of GSK2656157 or knockdown of PERK could increase the viability of BAY-885-treated BC cells (Figure 4b). Additionally, GSK2656157 and si-PERK attenuated apoptosis induced by BAY-885 in BC cells (Figure 4c). Western blot analysis showed that the cleavage of caspase-3, downregulation of Mcl-1, and upregulation of Bim caused by BAY-885 were abrogated by GSK2656157 or si-PERK in BC cells (Figure 4d). Collectively, these data suggested that BAY-885-induced ER stress was responsible for the downregulation of Mcl-1 and upregulation of Bim in BC cells (Figure 4e).

A. MCF-7 and MDA-MB-231 cells were treated with or without GSK2656157 for 4 h or transfected with si-NC or vector si-PERK for 24 h. Furthermore, the cells were treated with or without BAY-885 for another 24 h, and the expression of the indicated proteins was analyzed using western blot analysis. B. The cells were treated as described above, and cell viability was measured. C. Cell death was measured. D. The expression of the indicated proteins was measured using western blot analysis. E. A schematic diagram showing the BAY-885-induced apoptosis. Data are representative of at least three independent experiments; *P < 0.05; **P < 0.01.

## 4. Discussion

The MEK5/ERK5 axis, which can be activated in response to various signals such as stress, growth factors, and cytokines, is a part of the MAPK family [11]. Recently, many studies have suggested that MEK5/ERK5 contributes to tumorigenesis in BC. For example, the expression of ERK5 is much higher in BC cells than in adjacent normal cells and correlates with a poorer prognosis [12]. Additionally, MEK5/ERK5 signaling plays an essential role in the epithelial-to-mesenchymal transition process and metastasis of BC cells [13]. The MEK5/ERK5 axis represses estrogen receptor expression and promotes hormone-independent tumorigenesis in BC [14]. A recent study showed that inhibition of ERK5 enhanced the efficacy of anti-HER2 agent lapatinib in human breast cancer xenografts [15]. Thus, targeting the MEK5/ERK5 axis might be a potential strategy for the treatment of BC. BAY-885 is a novel ERK5 inhibitor, and its anti-tumor effects are rarely studied. In this study, we assessed the effects of BAY-885 on BC cells and its underlying mechanisms of action. Our data suggested that BAY-885 could be used as a promising therapeutic agent against BC.

The induction of apoptosis is a promising strategy for destroying cancer cells. In this study, it was found that BAY-885 reduced viability and induced apoptosis in BC cells, which was confirmed by the cleavage of caspase-3. This observation is in line with previous studies, which also reported that inhibition of ERK5 induced caspase-dependent apoptosis in colon cancer cells [16]. Although inhibition of ERK5 is a potent strategy to kill cancer cells, ERK5 kinase inhibitors often fail to recapitulate ERK5 genetic ablation phenotypes, and the efficiency is comprised in cancer cells [17]. In our study, upregulation of Bim and activation of ER stress were observed in BC cells after BAY-885 treatment. Those findings completely recapitulated the effect of ERK5 deletion on Bim and ER stress [18–20]. Additionally, we observed that genetically-induced activation of ERK5 abrogated the anti-tumor effects of BAY-885 in BC cells. Thus, our data suggest that BAY-885 is a potent and specific ERK5 inhibitor.

Combination therapy is a potent strategy to enhance the efficacy of chemotherapeutics and overcome chemoresistance in various cancers [21]. This led us to investigate whether BAY-885 has synergistic effects with different chemotherapeutic agents. Interestingly, we found that BAY-885 enhanced the apoptosis induced by various chemotherapeutic agents in BC cells. This finding was consistent with some studies, which reported that inhibition of ERK5 could overcome resistance, thereby enhancing the apoptosis induced by the EGFR inhibitor osimertinib in lung cancer cells [19,22]. Therefore, it would be interesting to test the effects of BAY-885 in combination with chemotherapeutic agents in BC clinically.

A key finding of our study is that Bim upregulation is vital for the apoptosis induced by BAY-885 in BC cells. To date, the relationship between ERK5 and Bim is still controversial. For instance, one study found that ERK5 activation led to the phosphorylation and degradation of Bim and thereby inhibited cell death during mitosis [23]. However, another study found that Bim phosphorylation is irrelevant to the activation of ERK5 [24]. Therefore, the relationship between ERK5 and Bim might be cell type- and/or stimuli-specific. More investigations are required to verify the correlation between ERK5 and Bim. Bim is a well-known tumor suppressor, and upregulation of Bim can overcome drug resistance in various cancers, including BC [25]. However, high levels of Bim might be a poor prognostic biomarker in patients with BC [26]. Thus, it would be interesting to examine the correlation between Bim expression and the clinical outcome of ERK5 inhibitors. Another important finding of our study was that BAY-885 inhibited the expression of Mcl-1 in BC cells. Previous studies reported that blockade of MEK1/2–ERK1/2 led to the downregulation of Mcl-1 in lung cancer cells [27,28]. However, information about the correlation between ERK5 and Mcl-1 is scarce. For the first time, our study revealed that blockade of ERK5 led to the downregulation of Mcl-1. High levels of Mcl-1 are frequently observed in patients with BC and are correlated with poor prognosis [29]. Mcl-1 is also critical for stem cell activity, and high Mcl-1 expression correlates with the expression of stemness markers in human BC cells [30]. Thus, targeting Mcl-1 is a promising strategy for BC treatment.

Evidence suggests that agents triggering ER stress can be applied as promising strategies to destroy BC cells. For example, betulinic acid could trigger ER stress-mediated apoptotic pathways in BC cells [31]. Another study found that oleandrin induced apoptosis in BC cells by activating ER stress [32]. ER stress induces a stress response via the UPR pathway. Three major sensors can regulate ER stress, namely, IRE1-α, ATF6, and PERK [33,34]. Activation of PERK can lead to the phosphorylation of downstream targets such as eIF2 α, ATF4, and CHOP [33]. In this study, RNA-sequencing revealed that BAY-885 treatment led to the activation of ER stress and URP pathways. Furthermore, western blot analysis showed that BAY-885 treatment led to the upregulation of p-PERK, ATF4, and CHOP. These findings suggested that BAY-885 could trigger ER stress in BC cells. To date, knowledge about the correlation between MEK5/ERK5 and ER stress is still limited. One study reported that deficiency of CHOP could ameliorate the ERK5 inhibition-mediated apoptosis caused by streptozotocin in pancreatic β cells [20]. The combination of dexmedetomidine and netrin-1 has been shown to decrease ER stress by activating ERK5, consequently preventing cerebral ischemia-reperfusion injury [35]. Another recent study also found that inhibition of ERK5 caused ER stress and thereby induced autophagy-medicated cell death in cancer cells [36]. In line with these studies, we revealed that BAY-885-induced ER stress and URP in BC cells. Furthermore, we found that forced expression of a constitutively activated form of MEK5 (MEK5DD) abrogated the ER stress induced by BAY-885. These findings provided novel insights into the crosstalk between MEK5/ERK5 and ER stress. Notably, we discovered that BAY-885-induced ER stress is required for the downregulation of Mcl-1 and the upregulation of Bim, as indicated by the use of an ER stress inhibitor (GSK2656157), and that genetic knockdown of PERK abolished BAY-885ʹs effects on Mcl-1 and Bim expression (Figure 4d). In accordance with our findings, many studies found that activation of ER stress caused downregulation of Mcl-1 [37–39]. Upregulation of Mcl-1 could attenuate the apoptosis induced by ER stress in melanoma cells [40]. A feedback loop between Mcl-1 and ER stress has also been identified [41]. Although previous studies found that inhibition of ERK5 induced apoptosis relied on the upregulation of Bim in different cells [18,19]. To the best of our knowledge, we revealed for the first time that blockage of ERK5 caused upregulation of Bim in an ER stress activation-dependent manner.

This study has some limitations. First, the anti-tumor effects of BAY-885 were only evaluated *in vitro*; therefore, it is worthwhile to investigate them in a xenograft mice model. Second, RNA sequencing results showed that BAY-885 affected other signaling pathways. It would be interesting to examine whether other signaling pathways are involved in the anti-tumor effects of BAY-885.

## 5. Conclusion

This study showed that BAY-885 exhibited selective cytotoxicity against BC cells but not normal cells. Combined treatment of BAY-885 and other chemotherapeutics synergistically induced apoptosis of BC cells. Mechanistically, BAY-885 induced apoptosis via the mitochondrial pathway in BC cells. Forced expression of Mcl-1 or silencing of Bim inhibited the apoptosis induced by BAY-885 in BC cells. Moreover, BAY-885 treatment induced ER stress, thereby leading to the downregulation of Mcl-1 and upregulation of Bim in BC cells.

Hence, our findings suggest that BAY-885 can be applied as a potent agent alone or in combination with other therapeutics for the treatment of BC.

## Data Availability

Data are available upon reasonable request to the corresponding author
